# An Assessment of ChatGPT’s Responses to Common Patient Questions About Lung Cancer Surgery: A Preliminary Clinical Evaluation of Accuracy and Relevance

**DOI:** 10.3390/jcm14051676

**Published:** 2025-03-01

**Authors:** Marina Troian, Stefano Lovadina, Alice Ravasin, Alessia Arbore, Aneta Aleksova, Elisa Baratella, Maurizio Cortale

**Affiliations:** 1 Thoracic Surgery Unit, Cattinara University Hospital, 34149 Trieste, Italy; 2 Thoracic Surgery Unit, Careggi University Hospital, 50134 Florence, Italy; 3 Department of Medical, Surgical and Health Sciences, University of Trieste, 34151 Trieste, Italy; aaleksova@units.it (A.A.);

**Keywords:** artificial intelligence (AI), ChatGPT, patient education, thoracic surgery, lung cancer, outpatient setting

## Abstract

**Background**: Chatbots based on artificial intelligence (AI) and machine learning are rapidly growing in popularity. Patients may use these technologies to ask questions regarding surgical interventions, preoperative assessments, and postoperative outcomes. The aim of this study was to determine whether ChatGPT could appropriately answer some of the most frequently asked questions posed by patients about lung cancer surgery. **Methods**: Sixteen frequently asked questions about lung cancer surgery were asked to the chatbot in one conversation, without follow-up questions or repetition of the same questions. Each answer was evaluated for appropriateness and accuracy using an evidence-based approach by a panel of specialists with relevant clinical experience. The responses were assessed using a four-point Likert scale (i.e., “strongly agree, satisfactory”, “agree, requires minimal clarification”, “disagree, requires moderate clarification”, and “strongly disagree, requires substantial clarification”). **Results**: All answers provided by the chatbot were judged to be satisfactory, evidence-based, and generally unbiased overall, seldomly requiring minimal clarification. Moreover, information was delivered in a language deemed easy-to-read and comprehensible to most patients. **Conclusions**: ChatGPT could effectively provide evidence-based answers to the most commonly asked questions about lung cancer surgery. The chatbot presented information in a language considered understandable by most patients. Therefore, this resource may be a valuable adjunctive tool for preoperative patient education.

## 1. Introduction

Lung cancer remains one of the most prevalent and lethal cancers worldwide, presenting significant challenges for patients, healthcare professionals, and researchers alike [1,2]. Surgical intervention is a critical topic for patients struggling with diagnosis and treatment, and it often leads to many questions regarding procedural details, recovery expectations, perioperative risks, and preoperative preparation [1,2,3]. As healthcare becomes increasingly digitalized, people have access to numerous internet resources that allow the quick and easy retrieval of information regarding health issues, possibly both aiding and hindering their decision-making process.

In this context, chatbots based on artificial intelligence (AI) and machine learning are rapidly growing in popularity and are opening new avenues for information dissemination and medical education [4,5,6,7]. Based on natural language processing (NLP) models, AI-driven chatbots can engage in meaningful conversations, provide responses to diverse queries, and may be used to ask questions regarding surgical interventions, preoperative assessments, and postoperative aspects [7,8,9,10,11].

However, the growing influence of AI applications in healthcare necessitates careful consideration, particularly with reference to the accuracy and reliability of the information they provide. It is true that recent advancements in AI have highlighted its expanding role in areas like intensive care and perioperative medicine, potentially improving outcomes by processing real-time data and providing personalized adjustments, as seen in cardiovascular procedures and pulmonary hypertension care [12,13,14]. Nevertheless, as the quality of AI-generated responses has yet to be rigorously evaluated, it is crucial to recognize that some information may be misleading or incorrect. Despite these concerns, AI chatbots could serve as valuable resources for addressing frequently asked questions (FAQs) in a medical context, thus potentially enhancing patient understanding and engagement [4,5,6,7,8,9,10,11,15,16].

In this study, we aimed to investigate the effectiveness of ChatGPT, version 3.5, a well-known AI-driven chatbot, in answering the most common questions patients have about lung cancer surgery. By evaluating the chatbot’s ability to provide accurate and informative responses, we tried to clarify its potential as a valuable tool for patient education in healthcare. Additionally, this analysis may help us understand the strengths and limitations of AI tools like ChatGPT in addressing lung cancer surgery queries, underscoring the necessity for reliable and supportive information to enhance patients’ engagement and knowledge, while mitigating the risks associated with misinformation.

## 2. Materials and Methods

ChatGPT, developed by OpenAI, is an advanced language model that utilizes AI to generate human-like text based on user input. Capable of addressing a variety of tasks, such as answering questions, providing explanations, and engaging in dialogue, ChatGPT is particularly noted for its subtle understanding of context and its ability to generate detailed and informative content [17].

To evaluate its effectiveness in addressing patient inquiries about lung cancer surgery, we conducted a thorough assessment of its responses to a selection of FAQs. The methodology comprised several key steps. First, we compiled a comprehensive list of common questions regarding lung cancer surgery, sourced from patient interviews, online health forums, and insights from medical professionals. Next, we utilized ChatGPT to generate responses to these FAQs, ensuring that the prompts were clearly framed to elicit relevant outputs. To gauge the appropriateness and accuracy of the chatbot’s answers, a four-point Likert scale was employed to assess the responses for clarity, correctness of medical facts, relevance to the posed question, and the potential for misinformation or oversimplification, as outlined in Table 1.

The Likert scale categorized responses into four levels: satisfactory responses which provided fundamentally accurate information; those requiring minimal clarification which were deemed correct but lacking detail; responses needing moderate clarification which contained outdated or irrelevant information; and responses requiring substantial clarification which were deemed incorrect or overly generalized, thus posing a risk for significant misinterpretation.

In addition to evaluating the quality of the chatbot’s responses, we also analyzed the word length of each answer to assess the risk of information overload. After generating responses for the 16 FAQs, we measured the number of words in each response to determine whether the length of the answers provided by ChatGPT was appropriate for patient understanding. Indeed, too long answers may lead to information overload, whereas too short answers could risk omitting important details, thus hindering comprehension.

The evidence-based nature of the chatbot’s responses was assessed by a panel of five thoracic surgeons with a minimum of 3 years of working experience following the completion of their training. All specialists had been actively working in the same clinical unit for the past 3 years, ensuring a shared understanding of current and local practice in lung cancer surgery. The panel compared the chatbot’s answers to authoritative, peer-reviewed literature and clinical guidelines pertaining to lung cancer surgery. The responses were evaluated for alignment with established best practices and the most current evidence available, ensuring that the information provided was scientifically accurate, up-to-date, and consistent with expert consensus in the field.

No ethics approval was required for the nature of this study.

## 3. Results

**Figure 1:**
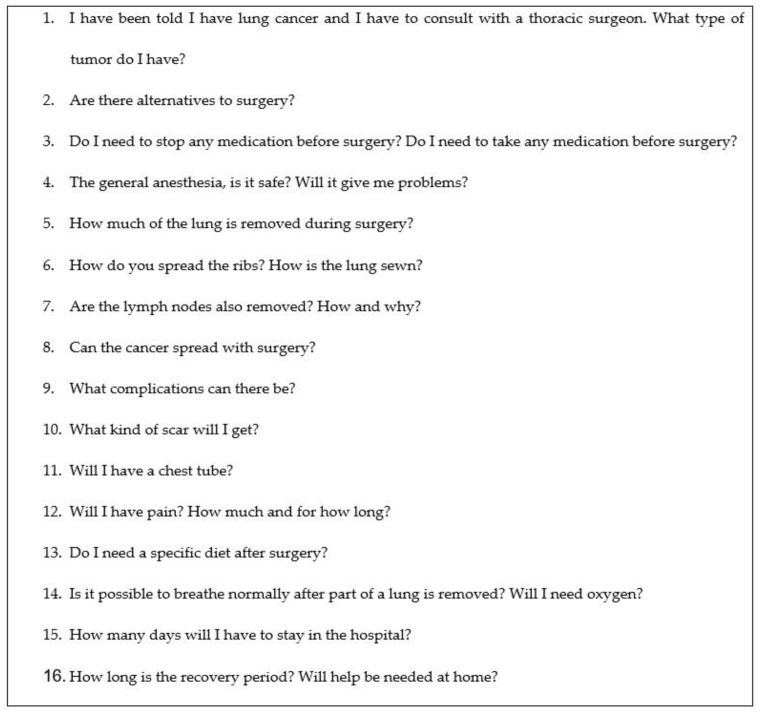
Selected FAQs about lung cancer surgery posed to ChatGPT.

A list of 16 FAQs asked by patients about lung cancer surgery were posed to ChatGPT version 3.5 in one conversation, without follow-up questions or the repetition of the same question, on 15 May 2024. The selected FAQs are reported in Figure 1.

The ChatGPT answers were evaluated by a panel of thoracic surgeons with relevant clinical experience using a four-point Likert scale. A sample of ChatGPT answers and their median evaluations are reported in Table 2. The full answers and evaluations to FAQs can be found in Appendix A.

**Figure 2:**
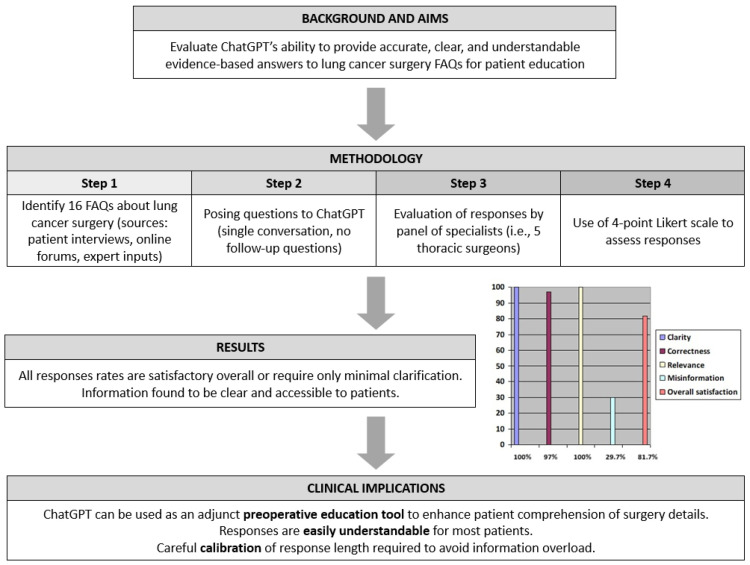
Summary of study aims, results, and conclusions.

All answers provided by the chatbot were judged by the panel to be satisfactory, evidence-based, and generally unbiased overall. Figure 2 provides a visual summary of the study aims, results, and conclusions.

In addition to evaluating the quality of the answers, we analyzed the word length of each response to assess the risk of information overload. The word counts for the 16 responses generated by ChatGPT ranged from 143 to 413 words, with a median word count of 323 words. The variation in response length reflects the complexity of the questions posed, with more intricate topics leading to longer answers. The distribution of word counts for each response are presented in Table 3.

While the word count varied across different FAQs, it is worth noting that no specific word length restriction was imposed on the model’s prompts. This allowed ChatGPT to generate responses of varying lengths based on the perceived complexity of each question. Although some answers exceeded 350 words, most were within a range deemed acceptable for patient education, with clear and concise explanations. However, the relatively high variability in word length suggests that, depending on the patient’s familiarity with medical concepts, some answers may be at risk of overwhelming individuals with excessive detail.

## 4. Discussion

In recent years, the integration of AI within healthcare practice has witnessed remarkable momentum and considerable expansion. This trend is largely driven by the recognition of AI’s potential to significantly improve various facets of healthcare, including diagnostics, treatment planning, and operational efficiency [4,5,6,12,13,14]. Notably, research exploring AI in critical illness management, such as cardiogenic shock, has shown considerable promise in enhancing clinical decision-making. Zymliński et al. [12] demonstrated how AI algorithms could optimize the use of percutaneous mechanical circulatory support devices, like the Impella, by providing real-time monitoring and personalized treatment adjustments to improve patient outcomes. Similarly, Consolo et al. [13] highlighted the role of AI in supporting the management of mechanical circulatory support in critically ill patients, emphasizing the importance of incorporating AI into complex clinical scenarios. Additionally, Attaripour Esfahani et al. [14] recently examined the application of AI in managing pulmonary hypertension. Their findings revealed that AI can analyze complex patient data to improve early detection and optimize treatment protocols, underscoring its significant potential in managing chronic and progressive diseases. All in all, these studies illustrate the broader potential for AI to enhance healthcare delivery.

In this context, there is also a burgeoning interest in utilizing AI technologies in the field of patient education to enhance their comprehension of health conditions and available treatment options, as well as to foster a more informed and proactive approach to healthcare [7,8,9,10,11,15,16].

In the present study, we evaluated ChatGPT answers to common patient questions about lung cancer surgery and we found that responses were generally satisfactory, evidence-based, and unbiased, thus suggesting the validity of AI-driven chatbots as a potential source for patient education. Moreover, the chatbot often recommended discussing any question with the referral healthcare team before reaching a final conclusion.

Our results are in line with previous literature findings. A recent study by Dai et al. [9] evaluated the effectiveness of ChatGPT as a potential assistant for thoracic surgeons. The researchers assessed AI’s ability to provide accurate and relevant information in response to clinical scenarios and questions related to thoracic surgery. Although human oversight may still be required to ensure the reliability of data, particularly in complex clinical situations, the study indicated that ChatGPT presented a commendable level of performance, offering answers that were generally accurate and informative. Similar conclusions were drawn by Ferrari-Light et al. [10], who investigated the quality and accuracy of ChatGPT-generated responses to common patient inquiries about lung cancer surgery. In this study, ChatGPT was found to effectively provide understandable and relevant data, suggesting its potential to act as a useful informational tool. However, the authors also emphasized the need for further research to ensure the reliability and safety of AI-generated medical information in clinical settings.

The role of AI-driven chatbots in answering common patient questions has also been explored in other medical fields. For example, Mika et al. [11] evaluated ChatGPT responses to common inquiries regarding total hip arthroplasty. The authors found that the chatbot provided accurate and relevant information in a way that most patients would be able to understand, thus concluding that AI may act as a valuable additional tool for patient education and understanding prior to surgical consultations. Similarly, Janopaul-Naylor et al. [15] demonstrated that AI-driven chatbots can also effectively address common patient questions regarding cancer. In their study, the authors indicated that these tools could serve as valuable adjunct to physician–patient communication, helping to clarify possible complex medical information and improve patients’ engagement in their healthcare journey. However, they once more underlined the importance of human oversight to ensure the accuracy and relevance of AI-generated responses, especially concerning cancer prognosis, to prevent misleading information and unnecessary emotional distress for patients and their families.

The appropriateness and comprehensiveness of AI responses were likewise evaluated by Shao et al. [8], who explored ChatGPT responses to a set of FAQs in thoracic surgery in different language contexts (i.e., English and Chinese). The study indicated that ChatGPT can provide responses of comparable quality in different languages, thus highlighting its potential in bridging communication gaps and ensuring that the information provided is accessible and appropriate to all users. These findings align with the present study, as ChatGPT answers were deemed effective in communicating complex medical information to a broad audience, potentially improving the understanding of medical issues among diverse patient populations.

Another interesting aspect of the present study is the analysis of the word length of ChatGPT’s responses, which we found to range between 142 and 413 words, with a median word count of 323. This variation in response length highlights the potential risk of information overload, a concern frequently discussed in the literature on patient consultations [18,19,20,21,22,23,24,25]. It is crucial to ensure that responses are not overly detailed, as patients may struggle to differentiate between essential and nonessential information during consultations. D’Andria Ursoleo et al. [18] recently emphasized the importance of clear and empathetic communication in alleviating patient’s anxiety and enhancing trust, which are vital components in improving surgical outcomes. The results from our study further suggest that the length and complexity of AI-generated responses should be carefully calibrated to avoid overwhelming patients with excessive information. Moreover, studies on perioperative care and patient consultations emphasize the importance of presenting information in a way that is both accessible and digestible, which can significantly improve patient understanding and compliance [19,20,21,22,23,24]. Therefore, future AI-based tools for patient education should incorporate adaptive features, allowing responses to be tailored in length and complexity according to individual patient needs and comprehension levels.

One potential limitation of our study is that the responses generated by the chatbot may not be identical when asked by different users or at different times. As we did not assess the consistency of the answers across various users, platforms, or time points, this introduces the possibility of variability in the chatbot’s responses. In addition, there may have been an interpretation bias based on the subjective interpretation of AI-generated responses by the interviewed specialists. Consequently, the evaluation of the chatbot’s answers in this study may not fully capture the extent to which the information provided would be consistent and reliable across different settings, which could introduce some bias in the reported observations.

Although our study provides a detailed evaluation of the chatbot’s answers, it is important to note that we did not conduct a patient-centered assessment to assess whether patients find these AI-generated responses helpful and acceptable. Further studies involving patient feedback would be valuable in determining the actual usefulness of this resource from a patient’s perspective. Additionally, the chatbot’s responses are generalized and may lack specificity regarding individual centers, patient demographics, or institutional practices. As patient care and outcomes can vary between institutions, the clinical applicability of these responses may be limited, and caution should be exercised when considering their use in specific healthcare settings.

Despite these limitations, the present study aligns with current literature data [8,9,10,11,12,13,14,15], underscoring the promising potential of ChatGPT as a tool for enhancing patient education in medical contexts. Its ability to provide timely and accessible information could significantly support patient engagement and shared decision-making, although it remains crucial for healthcare providers to keep on engaging in dialogue to ensure that patients receive accurate information, personalized care, and the opportunity to directly address any concerns or questions they may have.

## 5. Conclusions

The incorporation of AI-driven tools like ChatGPT into patient care offers both significant opportunities and challenges. In this study, we demonstrated that AI-chatbots can effectively deliver evidence-based responses to the questions most frequently asked by patients preparing for lung cancer surgery, presenting the information in language that is generally easily understandable. Being user-friendly and potentially accessible at no cost, these resources have the capability to serve as a valuable clinical tool for enhancing preoperative patient education.

However, the complexity and variability of individual patient cases require that healthcare providers should review chatbot-generated information to ensure its relevance and accuracy before it is used for patient education. Until further studies confirm the chatbot’s ability to consistently offer clinically appropriate, personalized information, human oversight remains essential. Moreover, it is important to recognize that AI may lack empathy and non-verbal clues, which are essential for fostering emotional reassurance and building a strong doctor–patient relationship. These elements, such as facial expressions and physical contact, play a crucial role in patient-centered care and, in our opinion, are irreplaceable by technology. Therefore, as AI tools like ChatGPT are integrated into clinical practice, it is essential to maintain a patient-centered approach, using AI as a supplement to, not a replacement for, traditional patient education. Healthcare providers should review and tailor AI-generated information to ensure its relevance to individual patients. Additionally, assessing patient preferences regarding the use of AI in their care will help refine these tools to better meet their needs, ensuring that the emotional and personalized aspects of healthcare are preserved. While AI has significant potential to support patient education, future studies are required to confirm its effectiveness and its impact on patient satisfaction, with human interaction remaining central to high-quality, patient-centered care.

## Figures and Tables

**Table 1:** ChatGPT response rating scale.

Likert Score	Accuracy Description
1	Strongly disagree, requires substantial clarification
2	Disagree, requires moderate clarification
3	Agree, requires minimal clarification
4	Strongly agree, satisfactory

**Table 2:** Sample of ChatGPT answers and their evaluation by clinical specialists.

Question	ChatGPT Answer	Likert-Scale Evaluation
Are there alternatives to surgery?	Yes, there are alternatives to surgery for treating lung cancer, depending on the type and stage of the cancer, as well as other individual factors such as overall health and personal preferences. Some common alternatives to surgery include: 1. Radiation therapy: Radiation therapy uses high-energy rays to kill cancer cells. It can be used as the main treatment or in combination with other treatments such as surgery or chemotherapy. Radiation therapy may be an option for patients who are not suitable candidates for surgery or who prefer non-invasive treatment. 2. Chemotherapy: Chemotherapy involves using drugs to kill cancer cells or stop them from growing. It can be used alone or in combination with other treatments like surgery or radiation therapy. Chemotherapy may be recommended for advanced lung cancer or as adjuvant therapy after surgery to reduce the risk of cancer recurrence. 3. Targeted Therapy: Targeted therapy drugs target specific genetic mutations or proteins that are involved in the growth and spread of cancer cells. These drugs can be effective in treating certain types of lung cancer, particularly non-small cell lung cancer with specific genetic mutations. 4. Immunotherapy: Immunotherapy works by boosting the body’s immune system to recognize and attack cancer cells. It has shown promising results in treating advanced lung cancer, particularly non-small cell lung cancer, and is often used as a first-line treatment or in combination with other therapies. 5. Clinical Trials: Clinical trials investigate new treatments or treatment combinations for lung cancer. Participating in a clinical trial may give you access to cutting-edge therapies that are not yet widely available. It’s essential to discuss all available treatment options with your healthcare team, including the potential benefits, risks, and side effects of each approach. They can help you make an informed decision based on your individual situation and preferences.	Clarity 4 Medical correctness 3 Relevance 4 Risk of misinformation 2
Is it possible to breathe normally after part of a lung is removed? Will I need oxygen?	After part of a lung is removed (a procedure known as lung resection), many people are able to breathe normally and lead active lives, especially if their lung function was good before the surgery. The remaining lung tissue is usually able to compensate for the lost tissue, allowing the body to maintain adequate oxygen levels and lung function. However, the extent to which lung function may be affected depends on various factors, including the size of the remaining lung tissue, the overall health of the individual, and any pre-existing lung conditions. Some people may experience a temporary decrease in lung function immediately after surgery due to post-operative pain and inflammation, but this typically improves over time as the body heals. In some cases, particularly if the remaining lung tissue is compromised or if there were pre-existing lung conditions, individuals may experience some degree of shortness of breath or reduced exercise tolerance after lung resection. However, many people are still able to perform daily activities and participate in physical exercise without significant limitations. In certain situations, such as if lung function is significantly compromised or if there are underlying lung conditions, supplemental oxygen therapy may be recommended after lung resection to help maintain adequate oxygen levels in the blood. This may be provided temporarily during the immediate post-operative period or on a long-term basis if necessary. It’s important to discuss any concerns about breathing or oxygen needs with your healthcare team before and after lung surgery. They can provide personalized guidance based on your individual circumstances and help you understand what to expect regarding breathing function and oxygen requirements after lung resection. Additionally, pulmonary rehabilitation programs may be available to help improve lung function and enhance overall respiratory health after surgery.	Clarity 4 Medical correctness 4 Relevance 4 Risk of misinformation 1

**Table 3:** Word count of ChatGPT’s responses.

Question	Topic	Word Count
1	Lung cancer diagnosis and tumor type	142
2	Surgical alternatives for lung cancer	300
3	Preoperative medication management	275
4	Risk and safety of general anesthesia	280
5	Extent of lung resection	304
6	Surgical techniques for lung cancer surgery	309
7	Lymph node removal in lung cancer surgery	334
8	Risk of cancer spread during surgery	312
9	Potential complications of lung cancer surgery	360
10	Postoperative scars and cosmetic outcomes	365
11	Postoperative chest tube placement	355
12	Postoperative pain management	381
13	Nutrition in lung cancer surgery	355
14	Respiratory function after lung resection	290
15	Length of hospital stay	342
16	Recovery time after lung cancer surgery	413

## Data Availability

All data underlying this article are available in the text.

## Abbreviations

The following abbreviations are used in this manuscript:

AI	Artificial Intelligence
NLP	Natural Language Processing
FAQs	Frequently Asked Questions

## Appendix A

In this appendix, we report ChatGPT’s full answers to the 16 FAQs and the evaluation of the answers by the panel of specialists.

**Question 1**. I have been told I have lung cancer and I have to consult with a thoracic surgeon. What type of tumor do I have?

**ChatGPT-3.5 answer**. I’m not a medical professional, but there are several types of lung cancer, and the specific type of tumor you have can vary. The two main categories of lung cancer are non-small cell lung cancer (NSCLC) and small cell lung cancer (SCLC). NSCLC accounts for about 80–85% of lung cancers and includes several subtypes such as adenocarcinoma, squamous cell carcinoma, and large cell carcinoma. SCLC is less common, but it tends to be more aggressive.

To determine the exact type of tumor you have, your doctor will likely order various tests, including imaging scans, biopsies, and possibly genetic tests. These tests will help them diagnose the type and stage of your lung cancer, which will then guide your treatment plan. It’s crucial to consult with your thoracic surgeon and other healthcare professionals to understand your diagnosis fully and explore your treatment options.

**Table A1:** Panel evaluation.

Clarity	Medical Correctness	Relevance	Misinformation
4	4	4	1

**Question 2**. Are there alternatives to surgery?

**ChatGPT-3.5 answer**. Yes, there are alternatives to surgery for treating lung cancer, depending on the type and stage of the cancer, as well as other individual factors such as overall health and personal preferences. Some common alternatives to surgery include:

1. **Radiation Therapy**: Radiation therapy uses high-energy rays to kill cancer cells. It can be used as the main treatment or in combination with other treatments such as surgery or chemotherapy. Radiation therapy may be an option for patients who are not suitable candidates for surgery or who prefer non-invasive treatment.
2. **Chemotherapy**: Chemotherapy involves using drugs to kill cancer cells or stop them from growing. It can be used alone or in combination with other treatments like surgery or radiation therapy. Chemotherapy may be recommended for advanced lung cancer or as adjuvant therapy after surgery to reduce the risk of cancer recurrence.
3. **Targeted Therapy**: Targeted therapy drugs target specific genetic mutations or proteins that are involved in the growth and spread of cancer cells. These drugs can be effective in treating certain types of lung cancer, particularly non-small cell lung cancer with specific genetic mutations.
4. **Immunotherapy**: Immunotherapy works by boosting the body’s immune system to recognize and attack cancer cells. It has shown promising results in treating advanced lung cancer, particularly non-small cell lung cancer, and is often used as a first-line treatment or in combination with other therapies.
5. **Clinical Trials**: Clinical trials investigate new treatments or treatment combinations for lung cancer. Participating in a clinical trial may give you access to cutting-edge therapies that are not yet widely available.

It’s essential to discuss all available treatment options with your healthcare team, including the potential benefits, risks, and side effects of each approach. They can help you make an informed decision based on your individual situation and preferences.

**Table A2:** Panel evaluation.

Clarity	Medical Correctness	Relevance	Misinformation
4	3	4	2

**Question 3**. Do I need to stop any medication before surgery? Do I need to take any medication before surgery?

**ChatGPT-3.5 answer**. Before surgery, it’s crucial to follow your surgeon’s instructions regarding medications. Here are some general guidelines:

- **Stop certain medications**: Some medications can increase the risk of bleeding during surgery or interact with anesthesia. Your surgeon will provide specific instructions on which medications to stop and when to stop them. These may include blood thinners (such as warfarin or aspirin), herbal supplements, and certain over-the-counter medications.
- **Take prescribed medications**: Depending on your medical history and the type of surgery you’re undergoing, your surgeon may instruct you to continue taking certain medications. These may include medications for chronic conditions like high blood pressure or diabetes. It’s essential to follow your surgeon’s instructions carefully and inform them of all the medications you’re currently taking.
- **Preoperative medications**: Your surgeon or anesthesiologist may prescribe medications to take before surgery to help manage pain, anxiety, or other symptoms. They may also prescribe antibiotics to reduce the risk of infection after surgery. Follow their instructions regarding the timing and dosage of these medications.
- **Avoid food and drink**: Your surgeon will likely instruct you to avoid eating or drinking anything for a certain period before surgery, typically starting the night before. This is to prevent complications such as aspiration during anesthesia.
- **Follow fasting guidelines**: Your surgeon will provide specific fasting guidelines, including when to stop eating and drinking before surgery. It’s essential to follow these guidelines closely to ensure a safe surgery and recovery.

Always communicate openly and honestly with your healthcare team about any medications you’re taking, including prescription drugs, over-the-counter medications, supplements, and herbal remedies. They can provide personalized guidance based on your individual medical history and the planned surgical procedure.

**Table A3:** Panel evaluation.

Clarity	Medical Correctness	Relevance	Misinformation
4	4	4	1

**Question 4**. The general anesthesia, is it safe? Will it give me problems?

**ChatGPT-3.5 answer**. General anesthesia is generally safe for most people when administered by trained professionals in a medical setting. However, like any medical procedure, it carries some risks and potential side effects. Before undergoing general anesthesia, your healthcare team will assess your medical history, current health status, and any risk factors to determine if it’s safe for you. Potential risks and side effects of general anesthesia may include:

- **Allergic reactions**: while rare, some people may have allergic reactions to the medications used in general anesthesia.
- **Nausea and vomiting**: Nausea and vomiting are common side effects of general anesthesia, particularly in the hours following surgery. Your anesthesia team can provide medications to help prevent or manage these symptoms.
- **Sore throat**: The breathing tube used during general anesthesia can cause a sore throat or irritation in the throat.
- **Confusion or memory loss**: Some people may experience confusion or memory loss after waking up from anesthesia, particularly in older adults.
- **Respiratory problems**: General anesthesia can temporarily affect breathing and lung function. Your healthcare team will monitor your breathing closely during surgery and provide support as needed.
- **Cardiovascular effects**: General anesthesia can affect blood pressure, heart rate, and other cardiovascular functions. Your anesthesia team will monitor your vital signs closely and provide medications or interventions to maintain stable cardiovascular function.
- **Rare complications**: In rare cases, general anesthesia can lead to more serious complications such as stroke, heart attack, or allergic reactions.

It’s essential to discuss any concerns or questions you have about general anesthesia with your healthcare team before surgery. They can provide detailed information about the risks and benefits and address any specific concerns based on your individual medical history and the planned surgical procedure.

**Table A4:** Panel evaluation.

Clarity	Medical Correctness	Relevance	Misinformation
4	4	4	1

**Question 5**. How much of the lung is removed during surgery?

**ChatGPT-3.5 answer**. The amount of lung removed during surgery depends on various factors, including the size and location of the tumor, the type and stage of lung cancer, and the overall health and lung function of the patient. The goal of lung cancer surgery is to remove the tumor along with a margin of healthy tissue to ensure that all cancerous cells are excised.

There are different types of lung cancer surgery, including:

- **Wedge resection**: In a wedge resection, the surgeon removes only a small wedge-shaped portion of the lung containing the tumor and a margin of healthy tissue around it. This procedure is typically used for small, early-stage tumors located near the surface of the lung.
- **Lobectomy**: A lobectomy involves removing one of the lobes of the lung. The lungs are divided into lobes (right lung has three lobes, and the left lung has two lobes), and removing a lobe may be necessary for larger tumors or those located centrally within a lobe.
- **Segmentectomy**: A segmentectomy involves removing a segment of the lung that contains the tumor. This procedure is less extensive than a lobectomy and may be suitable for smaller tumors located in specific segments of the lung.
- **Pneumonectomy**: A pneumonectomy is the removal of an entire lung. This procedure is less common but may be necessary for large tumors that involve an entire lung or for tumors located centrally.

The extent of lung resection required will be determined by your thoracic surgeon based on imaging studies, such as CT scans, and other diagnostic tests. The goal is to remove the cancerous tissue while preserving as much healthy lung tissue and function as possible. After surgery, your remaining lung tissue will compensate for the loss, and most people can lead a relatively normal life with one lung or a portion of one lung removed.

**Table A5:** Panel evaluation.

Clarity	Medical Correctness	Relevance	Misinformation
4	3	4	2

**Question 6**. How do you spread the ribs? How is the lung sewn?

**ChatGPT-3.5 answer**. In lung surgery, spreading the ribs and sewing the lung are both critical aspects of the procedure, aimed at providing access to the lungs and ensuring a secure closure after the tumor has been removed. Here’s a brief overview of these processes:

- **Spreading the ribs**: Rib spreading is typically performed using a rib spreader, also known as a rib retractor. This instrument is designed to gently separate and hold the ribs apart, creating space for the surgeon to access the lungs and perform the necessary procedures. The rib spreader is carefully inserted between the ribs at the incision site and gradually opened to widen the space between the ribs without causing damage to the surrounding tissues or nerves.

Spreading the ribs allows the surgeon to visualize and access the lungs, blood vessels, and other structures within the thoracic cavity more effectively, facilitating the removal of the tumor and any necessary repairs.

- **Suturing the lung**: After the tumor or affected portion of the lung has been removed, the remaining lung tissue may need to be sewn or stapled closed to ensure a secure closure and prevent air leaks or bleeding. The surgeon uses specialized sutures or surgical staples to close the incision in the lung tissue, taking care to create a watertight seal and minimize the risk of complications.

Depending on the extent of the surgery and the location of the incision, different suturing techniques may be used to achieve optimal closure and lung function. In some cases, additional procedures such as sealing the lung with a surgical sealant or applying tissue glue may be performed to reinforce the closure and enhance healing.

These steps are performed by highly skilled thoracic surgeons with extensive training and experience in lung surgery. The goal is to ensure a successful outcome while minimizing complications and preserving lung function to the greatest extent possible.

**Table A6:** Panel evaluation.

Clarity	Medical Correctness	Relevance	Misinformation
4	4	4	1

**Question 7**. Are the lymph nodes also removed? How and why?

**ChatGPT-3.5 answer**. Yes, in many cases, lymph nodes are removed during lung cancer surgery. The lymph nodes play a crucial role in the spread of cancer cells, as they can serve as pathways for cancer to metastasize (spread) to other parts of the body. Removing nearby lymph nodes allows the surgeon to assess whether the cancer has spread beyond the lung and helps determine the stage of the cancer.

Here’s why and how lymph nodes are removed during lung cancer surgery:

- **Staging**: Lymph node removal helps determine the stage of the cancer, which is essential for planning further treatment and predicting the prognosis. The stage of lung cancer is based on factors such as the size of the tumor, whether it has spread to nearby lymph nodes, and whether it has metastasized to other organs.
- **Assess Panel evaluation.Panel evaluation.ment of spread**: Examining the lymph nodes allows the surgeon to assess whether the cancer has spread beyond the lung. If cancer cells are found in the lymph nodes, it indicates a higher stage of the disease and may affect the choice of treatment options, such as chemotherapy or radiation therapy.
- **Prevention of recurrence**: Removing affected lymph nodes helps reduce the risk of cancer recurrence by eliminating potential reservoirs of cancer cells that could lead to the regrowth of the tumor or the spread of cancer to other parts of the body.

Lymph node removal during lung cancer surgery is typically performed using a procedure called lymphadenectomy or lymph node dissection. The surgeon carefully identifies and removes the lymph nodes located near the lungs and within the mediastinum (the area between the lungs), where lung cancer is most likely to spread.

The number and location of lymph nodes removed depend on factors such as the stage and type of lung cancer, as well as the surgical approach and the preferences of the surgical team. The goal is to remove enough lymph nodes to accurately assess the extent of the disease while minimizing the risk of complications and preserving overall lymphatic function.

**Table A7:** Panel evaluation.

Clarity	Medical Correctness	Relevance	Misinformation
4	4	4	2

**Question 8**. Can the cancer spread with surgery?

**ChatGPT-3.5 answer**. Surgery for lung cancer is primarily aimed at removing the tumor and any nearby affected tissue, including lymph nodes, in order to prevent the cancer from spreading further. However, there is a small risk that cancer cells may spread during surgery, particularly if the tumor is large or if there are complications during the procedure. Here are some factors to consider regarding the potential spread of cancer during surgery:

- **Inadvertent spread**: During surgery, there is a risk that cancer cells could be inadvertently released into the bloodstream or nearby tissues. This risk is generally minimized by careful surgical techniques, including the use of specialized instruments and procedures to contain and remove the tumor.
- **Seeding**: In some cases, surgery may cause cancer cells to spread to other parts of the body through a process known as “seeding”. This is rare but can occur if cancer cells are dislodged from the tumor and implant in nearby tissues or organs.
- **Intravasation**: Cancer cells may also spread through the bloodstream or lymphatic system during surgery, although this is less likely with lung cancer compared to other types of cancer.
- **Stage of cancer**: The risk of cancer spread during surgery may be higher for more advanced stages of lung cancer, where the tumor has grown larger or has spread to nearby tissues or lymph nodes.

It’s important to note that while there is a small risk of cancer spread during surgery, the benefits of surgery in terms of removing the primary tumor and reducing the risk of cancer recurrence generally outweigh the risks. Additionally, surgical techniques and protocols continue to evolve to minimize the risk of cancer spread and improve outcomes for patients undergoing lung cancer surgery.

Your healthcare team will discuss the risks and benefits of surgery with you in detail and provide personalized recommendations based on your individual circumstances and the stage of your cancer.

**Table A8:** Panel evaluation.

Clarity	Medical Correctness	Relevance	Misinformation
4	4	4	1

**Question 9**. What complications can there be?

**ChatGPT-3.5 answer**. Lung cancer surgery, like any major surgical procedure, carries certain risks and potential complications. While the likelihood of experiencing complications varies depending on factors such as the type of surgery, the stage of cancer, and the overall health of the patient, it’s essential to be aware of these potential risks. Some complications that may arise after lung cancer surgery include:

1. **Pneumonia**: Inflammation or infection of the lung tissue (pneumonia) can occur after surgery, particularly if there is a buildup of mucus or fluid in the lungs.
2. **Air leaks**: Air leaks from the lung or airways may occur after surgery, leading to pneumothorax (collapsed lung) or subcutaneous emphysema (air trapped under the skin).
3. **Bleeding**: While uncommon, bleeding can occur during or after surgery and may require additional procedures to control.
4. **Pulmonary embolism**: Blood clots that travel to the lungs (pulmonary embolism) can occur after surgery, particularly in patients who are at higher risk due to factors such as obesity, immobility, or a history of blood clots.
5. **Infection**: Surgical site infections or infections of the chest cavity (such as empyema) can occur after surgery and may require antibiotics or additional treatment.
6. **Fluid buildup**: Fluid may accumulate in the chest cavity (pleural effusion) after surgery, which may require drainage.
7. **Chronic pain**: Some patients may experience chronic pain or discomfort after surgery, particularly if nerves are damaged during the procedure.
8. **Cardiovascular complications**: Surgery can place stress on the cardiovascular system, leading to complications such as heart attack or arrhythmias (irregular heartbeats).
9. **Respiratory complications**: Lung surgery can affect breathing and lung function, leading to complications such as atelectasis (collapsed lung tissue) or respiratory failure.
10. **Anesthesia complications**: Complications related to anesthesia, such as allergic reactions or respiratory issues, can occur during or after surgery.

It’s important to discuss the potential risks and complications of lung cancer surgery with your healthcare team before undergoing the procedure. They can provide personalized information based on your individual circumstances and help you make informed decisions about your treatment options. Additionally, your healthcare team will monitor you closely during and after surgery to identify and manage any complications that may arise.

**Table A9:** Panel evaluation.

Clarity	Medical Correctness	Relevance	Misinformation
4	4	4	1

**Question 10**. What kind of scar will I get?

**ChatGPT-3.5 answer**. The type of scar you’ll have after lung cancer surgery can vary depending on several factors, including the surgical approach used, the size and location of the incision, and individual healing characteristics. Here are some common types of scars associated with different surgical approaches:

- **Traditional thoracotomy**: A traditional thoracotomy involves making a long incision between the ribs to access the lungs. This type of incision typically results in a noticeable scar along the side of the chest, which may be several inches long. The scar may appear raised or thickened initially but tends to fade and flatten over time.
- **Minimally invasive surgery (Video-Assisted Thoracoscopic Surgery—VATS)**: VATS is a minimally invasive surgical approach that uses small incisions and specialized instruments to access the lungs. The incisions are usually smaller than those used in a traditional thoracotomy and may result in smaller, less noticeable scars. These scars may be located on the chest wall or between the ribs and tend to heal relatively well with minimal scarring.
- **Robotic-assisted surgery**: Robotic-assisted surgery is another minimally invasive approach that uses robotic arms controlled by the surgeon to perform the procedure through small incisions. The scars associated with robotic-assisted surgery are similar to those of VATS and are typically smaller and less prominent than those of a traditional thoracotomy.
- **Sternotomy**: In some cases, particularly for more extensive lung surgeries or combined procedures involving other organs, a sternotomy (incision through the breastbone) may be necessary. This type of incision results in a scar along the center of the chest, which may be more noticeable than scars from lateral incisions.

Regardless of the type of incision used, it’s essential to follow your surgeon’s post-operative care instructions to promote optimal healing and minimize scarring. This may include keeping the incision clean and dry, avoiding strenuous activities, and protecting the incision from sun exposure.

While scars are a natural part of the healing process and may initially be red, raised, or itchy, they typically fade and become less noticeable over time. If you have concerns about scarring or wound healing, don’t hesitate to discuss them with your healthcare team. They can provide guidance and recommendations to help you achieve the best possible outcome.

**Table A10:** Panel evaluation.

Clarity	Medical Correctness	Relevance	Misinformation
4	4	4	1

**Question 11**. Will I have a chest tube?

**ChatGPT-3.5 answer**. Yes, it’s common for patients undergoing lung cancer surgery to have a chest tube placed after the procedure. A chest tube, also known as a thoracostomy tube or chest drain, is a flexible tube inserted into the chest cavity to drain air, blood, or fluid that may accumulate after surgery. Here’s why a chest tube may be necessary and what to expect:

- **Preventing pneumothorax**: During lung surgery, the lung may be partially or fully collapsed to facilitate the surgical procedure. After surgery, a chest tube may be inserted to re-expand the lung and prevent a condition called pneumothorax, where air accumulates in the pleural space around the lung, causing it to collapse.
- **Removing fluid**: Surgery can cause fluid to accumulate in the chest cavity, a condition known as pleural effusion. A chest tube allows the drainage of this fluid, reducing the risk of complications such as infection or impaired lung function.
- **Draining blood**: In some cases, there may be bleeding in the chest cavity after surgery, either from the surgical site or from nearby blood vessels. A chest tube can be used to drain this blood and prevent the formation of a hematoma (collection of blood) that could compress the lung or other structures.
- **Monitoring and treatment**: The presence of a chest tube allows healthcare providers to monitor drainage and assess lung function after surgery. It also provides a means for administering medications or other treatments directly into the chest cavity if necessary.

While having a chest tube can be uncomfortable, particularly in the immediate post-operative period, it plays a crucial role in the recovery process and helps promote optimal healing. Your healthcare team will monitor the chest tube closely and may remove it once drainage has decreased to a certain level and lung function has improved sufficiently.

It’s essential to follow your healthcare team’s instructions regarding care and management of the chest tube, including keeping the insertion site clean and dry, monitoring drainage output, and reporting any signs of infection or other complications promptly. Once the chest tube is removed, you’ll likely experience improved comfort and mobility as you continue to recover from surgery.

**Table A11:** Panel evaluation.

Clarity	Medical Correctness	Relevance	Misinformation
4	4	4	1

**Question 12**. Will I have pain? How much and for how long?

**ChatGPT-3.5 answer**. Pain is a common experience after lung cancer surgery, but the severity and duration of pain can vary from person to person and depend on factors such as the type of surgery, individual pain tolerance, and overall health. Here’s what to expect regarding pain after lung cancer surgery:

- **Immediate post-operative period**: In the immediate aftermath of surgery, you can expect to experience some degree of pain or discomfort, particularly at the incision site and in the chest area. The intensity of pain during this period is often highest, as the body is still recovering from the surgical trauma. Pain may be managed with pain medications administered by your healthcare team, including intravenous (IV) medications or patient-controlled analgesia (PCA) systems.
- **Days to weeks after surgery**: Pain typically decreases gradually in the days and weeks following surgery as the body heals. However, you may still experience some discomfort, particularly with movements such as coughing, deep breathing, or changes in position. Your healthcare team will prescribe pain medications, such as oral opioids or nonsteroidal anti-inflammatory drugs (NSAIDs), to help manage pain as needed during this recovery period.
- **Recovery and rehabilitation**: As you progress through the recovery process, pain should continue to improve, and you may gradually reduce or discontinue pain medications under the guidance of your healthcare team. Engaging in activities such as walking and breathing exercises can help promote healing and reduce discomfort over time.
- **Chronic pain**: While most patients experience a significant reduction in pain as they recover from surgery, some individuals may continue to experience chronic pain or discomfort in the chest or incision site. Chronic pain after lung cancer surgery is relatively uncommon but can occur due to factors such as nerve damage, scar tissue formation, or musculoskeletal issues. If you experience persistent or worsening pain, it’s essential to discuss it with your healthcare team, as they can provide additional evaluation and treatment options to help manage your symptoms.

Overall, while pain is a normal part of the recovery process after lung cancer surgery, it should gradually improve over time as you heal. It’s important to communicate openly with your healthcare team about your pain levels and any concerns you may have, as they can provide personalized guidance and support to help you manage pain effectively and optimize your recovery.

**Table A12:** Panel evaluation.

Clarity	Medical Correctness	Relevance	Misinformation
4	4	4	1

**Question 13**. Do I need a specific diet after surgery?

**ChatGPT-3.5 answer**. After lung cancer surgery, maintaining a healthy diet is essential to support healing, promote recovery, and optimize overall health. While there may not be a specific diet plan that applies to every individual post-surgery, there are general dietary guidelines and recommendations that can help facilitate the healing process and prevent complications. Here are some dietary considerations after lung cancer surgery:

- **Balanced nutrition**: Focus on consuming a balanced diet that includes a variety of nutrient-rich foods, such as fruits, vegetables, whole grains, lean proteins, and healthy fats. Adequate nutrition is crucial for supporting tissue repair, maintaining energy levels, and supporting immune function.
- **Adequate protein intake**: Protein is essential for wound healing and tissue repair. Aim to include protein-rich foods in your diet, such as lean meats, poultry, fish, eggs, dairy products, legumes, nuts, and seeds.
- **Hydration**: Stay well-hydrated by drinking plenty of fluids, such as water, herbal teas, broth-based soups, and electrolyte-containing beverages. Proper hydration is important for maintaining hydration status, preventing constipation, and supporting overall health.
- **Fiber-rich foods**: Include fiber-rich foods in your diet to help prevent constipation, which can be a common side effect of pain medications and reduced physical activity post-surgery. Good sources of fiber include fruits, vegetables, whole grains, legumes, and nuts.
- **Avoiding gas-producing foods**: Some foods may cause gas and bloating, which can be uncomfortable, particularly after abdominal or thoracic surgery. Avoiding gas-producing foods such as beans, cruciferous vegetables (e.g., broccoli, cauliflower, cabbage), and carbonated beverages may help reduce discomfort.
- **Small, frequent meals**: Eating smaller, more frequent meals throughout the day may be easier to tolerate than large meals, particularly if you experience nausea, appetite changes, or difficulty swallowing after surgery.
- **Vitamin and mineral supplementation**: Depending on your individual needs and nutritional status, your healthcare team may recommend vitamin and mineral supplements to ensure adequate intake of essential nutrients during the recovery period.

It’s essential to follow any specific dietary instructions provided by your healthcare team and to communicate any concerns or changes in appetite or digestion with them. They can provide personalized guidance and support to help you meet your nutritional needs and optimize your recovery after lung cancer surgery.

**Table A13:** Panel evaluation.

Clarity	Medical Correctness	Relevance	Misinformation
4	4	4	1

**Question 14**. Is it possible to breathe normally after part of a lung is removed? Will I need oxygen?

**ChatGPT-3.5 answer**. After part of a lung is removed (a procedure known as lung resection), many people are able to breathe normally and lead active lives, especially if their lung function was good before the surgery. The remaining lung tissue is usually able to compensate for the lost tissue, allowing the body to maintain adequate oxygen levels and lung function.

However, the extent to which lung function may be affected depends on various factors, including the size of the remaining lung tissue, the overall health of the individual, and any pre-existing lung conditions. Some people may experience a temporary decrease in lung function immediately after surgery due to post-operative pain and inflammation, but this typically improves over time as the body heals.

In some cases, particularly if the remaining lung tissue is compromised or if there were pre-existing lung conditions, individuals may experience some degree of shortness of breath or reduced exercise tolerance after lung resection. However, many people are still able to perform daily activities and participate in physical exercise without significant limitations.

In certain situations, such as if lung function is significantly compromised or if there are underlying lung conditions, supplemental oxygen therapy may be recommended after lung resection to help maintain adequate oxygen levels in the blood. This may be provided temporarily during the immediate post-operative period or on a long-term basis if necessary.

It’s important to discuss any concerns about breathing or oxygen needs with your healthcare team before and after lung surgery. They can provide personalized guidance based on your individual circumstances and help you understand what to expect regarding breathing function and oxygen requirements after lung resection. Additionally, pulmonary rehabilitation programs may be available to help improve lung function and enhance overall respiratory health after surgery.

**Table A14:** Panel evaluation.

Clarity	Medical Correctness	Relevance	Misinformation
4	4	4	1

**Question 15**. How many days will I have to stay in the hospital?

**ChatGPT-3.5 answer**. The length of hospital stay after lung cancer surgery can vary depending on several factors, including the type of surgery performed, the extent of the procedure, your overall health, and how well you recover post-surgery. Here’s a general overview of the typical hospital stay for different types of lung cancer surgery:

- **Minimally invasive surgery (VATS or robotic-assisted surgery)**: Patients undergoing minimally invasive lung cancer surgery often have shorter hospital stays compared to traditional open surgery. Depending on factors such as the extent of the surgery and your overall health, you may stay in the hospital for 2 to 4 days on average.
- **Traditional open surgery (thoracotomy)**: Patients undergoing traditional open lung cancer surgery, such as a thoracotomy, typically have longer hospital stays compared to minimally invasive approaches. Hospital stays for open lung surgery can range from 4 to 7 days or more, depending on factors such as the complexity of the surgery and your post-operative recovery.
- **Extent of surgery**: The extent of the lung resection and whether additional procedures are performed (such as lymph node removal or combined surgeries involving other organs) can also influence the length of hospitalization. More extensive surgeries may require a longer recovery period and hospital stay.
- **Post-operative recovery**: Your hospital stay will also depend on how well you recover from surgery and how quickly you meet certain milestones, such as being able to tolerate oral intake, manage pain, and mobilize safely. Your healthcare team will monitor your progress closely and determine when it’s appropriate for you to be discharged from the hospital.

It’s important to follow your healthcare team’s post-operative care instructions and attend any follow-up appointments as scheduled. After discharge from the hospital, you may require additional support and monitoring during the early stages of your recovery, which may include home care services, rehabilitation, and outpatient appointments.

Your healthcare team will provide personalized guidance and support throughout your hospital stay and recovery process, ensuring that you receive the necessary care and assistance to optimize your recovery and achieve the best possible outcome after lung cancer surgery.

**Table A15:** Panel evaluation.

Clarity	Medical Correctness	Relevance	Misinformation
4	4	4	1

**Question 16**. How long is the recovery period? Will help be needed at home?

**ChatGPT-3.5 answer**. The recovery period after lung cancer surgery can vary from person to person, depending on factors such as the type of surgery performed, the extent of the procedure, your overall health, and how well you respond to treatment. While the acute recovery phase typically lasts several weeks to months, it may take longer to fully regain strength and resume normal activities. Here’s what to expect during the recovery period:

- **Immediate post-operative period (hospital stay)**: The immediate post-operative period typically involves staying in the hospital for several days to a week, depending on the type of surgery and your post-operative recovery. During this time, you’ll be monitored closely by healthcare professionals, receive pain management, and gradually resume activities such as walking and deep breathing exercises.
- **Home recovery**: After discharge from the hospital, you’ll continue your recovery at home. The duration of home recovery can vary but may last several weeks to months, depending on factors such as the extent of the surgery and your overall health. During this time, you may experience fatigue, pain, and limitations in physical activity as your body continues to heal.
- **Follow-up appointments**: You’ll have regular follow-up appointments with your healthcare team to monitor your progress, assess wound healing, and address any concerns or complications that may arise. These appointments may include visits with your surgeon, oncologist, pulmonologist, or other healthcare providers involved in your care.
- **Rehabilitation and physical therapy**: Depending on your individual needs and recovery progress, your healthcare team may recommend pulmonary rehabilitation or physical therapy to help improve lung function, strength, and endurance. These programs may include exercises, breathing techniques, and education on managing symptoms and optimizing recovery.
- **Assistance at home**: Depending on the extent of the surgery and your level of mobility, you may require assistance with activities of daily living (ADLs) and household tasks during the early stages of your recovery. This assistance may come from family members, friends, or home care services, such as visiting nurses or aides.
- **Gradual return to normal activities**: As you progress through the recovery period, you’ll gradually resume normal activities and responsibilities. It’s essential to listen to your body, pace yourself, and avoid overexertion as you regain strength and endurance.

It’s important to follow your healthcare team’s post-operative care instructions, attend all follow-up appointments, and communicate any concerns or changes in symptoms promptly. Your healthcare team can provide personalized guidance and support to help you navigate the recovery process and achieve the best possible outcome after lung cancer surgery.

**Table A16:** Panel evaluation.

Clarity	Medical Correctness	Relevance	Misinformation
4	4	4	1
